# Real-time audio and visual display of the Coronavirus genome

**DOI:** 10.1186/s12859-020-03760-7

**Published:** 2020-10-02

**Authors:** Mark D. Temple

**Affiliations:** School of Science, Western Sydney University, Campbelltown Campus, Locked Bag 1797, Penrith South DC, NSW 1797 Australia

**Keywords:** Sonification, RNA sequence, Auditory display, Molecular animation, SARS-CoV-2, COVID-19

## Abstract

**Background:**

This paper describes a web based tool that uses a combination of sonification and an animated display to inquire into the SARS-CoV-2 genome. The audio data is generated in real time from a variety of RNA motifs that are known to be important in the functioning of RNA. Additionally, metadata relating to RNA translation and transcription has been used to shape the auditory and visual displays. Together these tools provide a unique approach to further understand the metabolism of the viral RNA genome. This audio provides a further means to represent the function of the RNA in addition to traditional written and visual approaches.

**Results:**

Sonification of the SARS-CoV-2 genomic RNA sequence results in a complex auditory stream composed of up to 12 individual audio tracks. Each auditory motive is derived from the actual RNA sequence or from metadata. This approach has been used to represent transcription or translation of the viral RNA genome. The display highlights the real-time interaction of functional RNA elements. The sonification of codons derived from all three reading frames of the viral RNA sequence in combination with sonified metadata provide the framework for this display. Functional RNA motifs such as transcription regulatory sequences and stem loop regions have also been sonified. Using the tool, audio can be generated in real-time from either genomic or sub-genomic representations of the RNA. Given the large size of the viral genome, a collection of interactive buttons has been provided to navigate to regions of interest, such as cleavage regions in the polyprotein, untranslated regions or each gene. These tools are available through an internet browser and the user can interact with the data display in real time.

**Conclusion:**

The auditory display in combination with real-time animation of the process of translation and transcription provide a unique insight into the large body of evidence describing the metabolism of the RNA genome. Furthermore, the tool has been used as an algorithmic based audio generator. These audio tracks can be listened to by the general community without reference to the visual display to encourage further inquiry into the science.

## Background

Modern computers have had a great impact on biological experimentation and data analyses to reveal otherwise hidden patterns in complex data. This is apparent in the field of genomic data analyses. The viral genome of the first patient suffering from COVID-19 was submitted to GenBank [1] on 5 January 2020 some weeks after the first patient had been hospitalised in December 2019 [2]. Within 4 months over 4.7 million people worldwide had tested positive to the virus and the disease referred to as COVID-19 with approximately 315,000 deaths reported by Johns Hopkins University [3]. During this time a large body of evidence has arisen regarding RNA sequence homology to other SARS like virus strains [4, 5] and these studies may help identify targets for immune recognition.

**Fig. 1:**
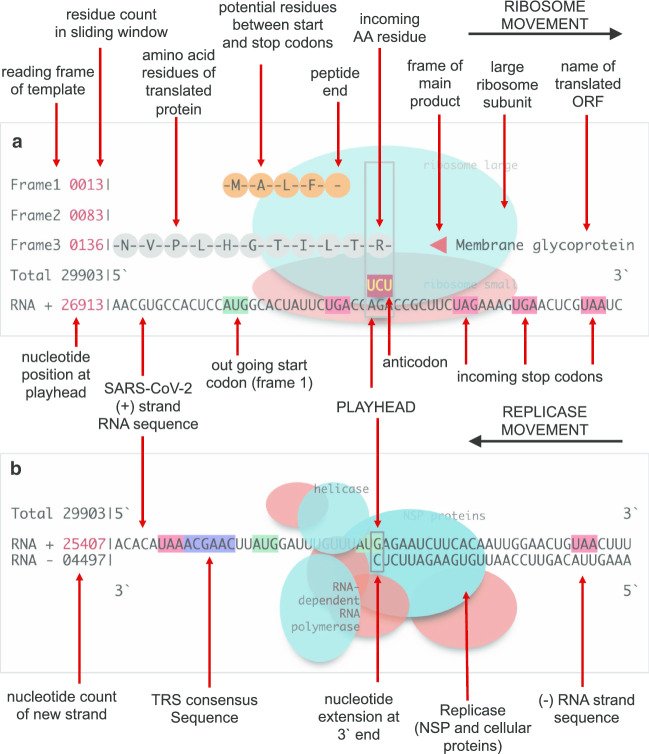
The animated display. Panel A shows the sliding window of the animated display in translation mode. Key features of the animated display are labelled such as the translated peptide sequences and the frame in which they occur, the presence of start and stop codons are highlighted in green and red, respectively. The location of the audio play-head is represented to coincide with the peptidyl-transferase centre of the ribosome. The sonified audio is generated as the SARS-CoV-2 genome sequence passes through the play-head. The direction in which the ribosome moves relative to the RNA sequence is indicated. Panel B shows the animated display in transcription mode. The newly synthesised minus RNA strand is shown below the genome sequence with the 3′ extended nucleotide shown in the play-head. The direction in which the replicase protein complex moves in relation to genome sequence is indicated

This paper demonstrates that sonification of RNA sequence data may also be useful to understand how the genome functions. The audio is generated using two approaches. The rules governing gene expression have been applied to the process of generating a linear audio stream similar to the expression of a linear sequence of amino acids. These methods are based on our previous approach to sonify DNA sequences [6]. These methods have been improved upon to include multi-layering of related audio tracks, and the inclusion of audio that is representative of sequence metadata. Additionally, a real time animated display (as shown in Fig. 1) of both the biological process and the notes being generated has been implemented. These displays are important since the ability to sequence DNA has vastly outpaced tools for their visualisation [7]. The real-time visual animation is an important addition since with sonification data alone is it difficult to relate the auditory display to the underlying sequence information. The combination of the auditory and visual displays is more informative than either display in isolation. In these displays the auditory and visual output are produced by the same events, since the sequence is processed in a linear fashion, and it is thought that the multisensory integration improves the perception of each [8].

The systematic and reproducible representation of data as sound is increasingly becoming a adjunct to the traditional visualization techniques of data inspection and analysis [9, 10]. In recent years auditory displays have become more popular to represent complex biological phenomena. A systematic review of over 150 sonification project highlighted the importance of pitch and spatial auditory dimensions in the auditory display [11]. Within the domain of molecular biology the properties of amino acid residues [12] and protein folding [13] have been sonified by a combination of musical techniques and sound effects. More recently researchers have generated musical scores representing amino acid sequences of protein structures and note sequences from short amino acid sequences [14]. Recently these authors applied their approach to sonifying the amino acid sequence and structure of the spike protein of SARS-CoV-2.

Genomic data has also provided a good candidate for sonification. These studies include sonification of the spectral properties of DNA, molecular analyses [15, 16] and a preliminary investigation into RNA structures [17, 18]. Gene expression data has been sonified to discriminate between differentially expressed genes [19, 20] and chip-seq data [21]. In the realm of cancer progression, epigenomic data has been sonified to investigate the importance of methylation [22]. It has also been suggested that audio may be useful to interpret tomography of human adipose and tumor tissue samples [23]. Microbial ecology data has been sonified into musical style by mapping rows of numerical data to chords [24, 25], towards the end of communicating complex results to people not specialised in the field.

Previous studies into DNA sonification for sequence analyses [6] demonstrated that mutations in repetitive DNA sequences such as telomer or alphoid DNA could be detected by ear alone and that coding regions could be distinguished from non-coding regions. The SARS-CoV-2 RNA genome does not contain extensive repetitive sequences except for the 3′ poly-A tail, hence this sequence provided more of a challenge for display. Given that the RNA genome is almost 30,000 kb in length it would be abrasive and fatiguing to the ear to use harsh or dissonant tones for the entire auditory display. Hence the decision was made to use more musical tones to generate the audio.

## Implementation

The web tool described in this paper [26] operates in two modes that broadly represents translation or transcription. The audio display is generated using algorithms based on biological rules to generate sound at the play-head. The play-head substitutes for a ribosome during translations mode or the RNA replicase/polymerase during transcription mode. A complex auditory stream was generated by overlaying up to 12 layers of audio (as summarised in Table 1). Each layer of audio is derived from an RNA motif directly or metadata was used to flag the region of sequence to be sonified. Additionally, prior to the start of each gene sequence an ascending run of 8 notes is triggered. This scale pattern is independent of the raw sequence data and is based solely on metadata relating to sequence position. This provides an audio cue to anticipate the upcoming gene coding sequence.

**Table 1:** The mapping of each RNA feature into a layer of the auditory display

Description	RNA feature	Note range	When is the feature sonified
As the sequence is processed each is sonified to create a constant audio stream	Nucleotide	4	Throughout the genome
	Di-nucleotide	16	Throughout the genome
	GC Content (10 bp)	10	Throughout the genome
	GC Content (100 bp)	10	Throughout the genome
Three of the same nucleotide repeats	Example: the poly-A tail	4	Anytime when condition is true
Codons (translation only)	Codon Frame 1	20	Between start and stop codons
	Codon Frame 2	20	Between start and stop codons
	Codon Frame 3	20	Between start and stop codons
Trinucleotides (transcription only)	Only the 1st and 3rd nucleotides are considered	16	Throughout the genome
Untranslated regions	Intragenic UTR regions (excluding 5′ and 3′ UTRs)	16	At genomic regions defined by GenBank metadata [2] Individual nucleotides were mapped to higher octaves ranges for the sake of audio clarity
Transcription regulating sequences (TRS)	Each nucleotide in TRS1 through to TRS10	16	
Polyprotein cleavage sites (translation only)	Nucleotides that code for the cleaved AA residues	4	
Stem and Loop regions (SL)	Each nucleotide in the identified region	16	

The most fundamental building block of RNA is an individual nucleotide and these were sonified as one of four individual notes whereas di-nucleotides were sonified as one of 16 notes and together these were panned left and right in the auditory display. Another characteristic of nucleic acid sequences which is often used as a metric of genome status is the GC content which is often represented as a ratio. Typically in Coronavirus the count of U is above average and C is below average whereas A is preferred over G [27] leading to a relatively low GC ratio. In our approach two GC ratios were determined within a sliding window of 10 or 100 nucleotides respectively across the entire genome. Each time the GC ratio changed by an increment or decrement of 0.1 a note was generated and these were panned against each other in stereo. When there is no change between two adjacent features in an audio stream, the first instance of the feature was allowed to play for a longer period of time rather than generating another instance of the same note. This approach provides a brief pause in the audio layer and provides an opportunity for another layer to be distinguished in the auditory display. Together these four audio tracks create an audio landscape that can be heard across the entire auditory display of the genome. These RNA features are not specific to either transcription or translation nor are they specific to a particular region of the genome. Other sonified genome features were layered over this sonified landscape.

In the translation mode, codons represent an important feature of RNA and these were sonified as 20 notes when representing translation into amino acids. No distinction was made between the start methionine or that which occurs in the body of the peptide sequence. Additionally, stop codons were sonified as an additional note since these are highly significant in the function of the genome. Overlapping codons in each of the three reading frames were sonified during translation to detect ORF’s in either frame. An important consideration in the modelling of translation was to use the start and stop codons in each reading frame to trigger or halt the audio derived from other codons. Additionally, in the visual display the audible codons were shown using the one letter amino acid representation. Using this simple method all gene sequences reported in the GenBank metadata were accurately represented in both the audio and visual displays. Additionally, all open reading frames throughout the RNA genome are shown and sonified. However, only open reading frames that correlate with the known metadata (gene sequences) were labelled in the visual display. This is consistent with prior approaches of mapping either individual bases [28], codons [29] or amino acids [30, 31] to musical notes in a manner inspired by the genetic code or codon usage during translation.

In the display representing transcription, codons per se were not considered. Instead tri-nucleotide features were considered for sonification, however, these were considered to be positioned adjacent in the sequence rather than overlapping. Given that there are 64 different tri-nucleotides it is not possible to use a traditional scale. A traditional piano consists of 7 octaves plus a minor third (88 notes). Given that there are 7 scale notes in an octave it would require over 9 octaves to accommodate 64 trinucleotides. Using synthesised notes could overcome this limitation but this would entail playing shrill high pitched notes that would be grating to the ear. Therefore, linear mapping of 64 codons to individual notes was avoided. In the transcription display, tri-nucleotides were mapped to 16 individual notes since only the first and third position in each was considered. Since trinucleotides play no functional role in the process of transcription there was no loss of information content using this approach and the audio could be designed to complement the single nucleotides and di-nucleotides in the audio stream and avoid the mapping to shrill notes. Additionally, tri-nucleotides were not mapped to start or stop functionality and these are audible throughout the entire genome. Their occurrence had no further effect in the auditory display.

Metadata specific to the Coronavirus SARS-CoV-2 sequence was used to supplement the audio generated from the intrinsic characteristics of the RNA sequence. Audio from un-translated sequences between the open reading frames were mapped to an audio stream at a reduced tempo so that they were more clearly distinguished from the coding regions. Additionally, the viral genome is known to contain 10 transcriptional regulatory sequences (TRS) and five known stem loop (SL) structures known to play a role in the function of the genome [32] and their occurrence was sonified. These conserved motifs were sonified and since they often occurred in the untranslated regions the audio from these two were panned in stereo.

The genome codes for a large polyprotein from a large open reading frame. This polyprotein is thought to be cleaved into 16 individual polypeptides (often referred to as NSP proteins) and the occurrence of the known cleavage sites was sonified. In addition to generating a short burst of notes, cleavage regions were also used to pause the progression of the play-head for a second or so by slowing the tempo to one tenth of the coding tempo. This effectively highlights the transition from one NSP sequence to the next. The occurrence of three or more identical nucleotides was also sonified since these are easy to detect by eye and their sound may help the user to keep track of where they are in the display.

Audio generated from each of these sequence motifs and metadata were combined to create a complex auditory display to represent either transcription or translation. As the audio is played a sliding window of 60 nucleotides is shown on the screen. At any point in time the first nucleotide in the visual play-head can be heard in the auditory display. Other sequence features are determined relative to the position of this nucleotide.

To play the entire genome takes approximately 96 min in translation mode which corresponds to approximately five nucleotides per second. This is slower than cellular translation which is thought to proceed at approximately 30 nucleotides per second [33], however, to play this any faster makes it difficult to interpret due to the shortened duration between each note and a different algorithm would need to be devised. In transcription mode the full display lasts 120 min since the number of nucleotides played per second is a little slower, this approach was taken to clearly distinguish it from translation mode.

Three sets of interactive buttons (summarised in Table 2) have been provided for each sonified feature so that each can be selected directly, for example a gene sequence or TRS can be selected and played directly without having to play through the proceeding sequence data. These buttons change to a red colour as the respective feature is displayed.

**Table 2:** Description of the navigation buttons from where users can begin playing the audio and visual displays

*Button set 1*	*RNA features associated with coding regions*
5′UTR	5′ untranslated region
Poly-/-protein	Two buttons representing the coding region before and after the -1 frameshift position of the large polyprotein
9 U regions	Each navigates to an untranslated region between ORF’s
-S-	Region coding for the canonical S protein
-E-	Region coding for the canonical E protein
-M-	Region coding for the canonical M protein
-N-	Region coding for the canonical N protein
ORF 3a, ORF 6, ORF 7a, ORF 7b, ORF 8, ORF 10	Regions thought to code for other proteins or polypeptides
3′UTR	5′ untranslated region
*Button set 2*	*RNA features associated with the NSP proteins*
5′UTR	5′ untranslated region
N1—N16	Location of the 16 NSP proteins within the large polyprotein
14 C sites	Cleavage sites within the translated polyprotein giving rise to the 16 individual NSP proteins
S—ORF 10	Region of the RNA sequence downstream of the polyprotein
3′UTR	5′ untranslated region
*Button set 3*	*RNA features associated with the TRS regions*
5′UTR	5′ untranslated region
T1—T10	Location of TRS 1 to TRS 10. TRS1 is sometimes referred to as the leader TRS and is linked to the subsequence TRS 2—10 to produce the sub-genomic regions during transcription
5 SL regions	Stem Loop regions giving rise to structured regions of RNA. These are formed due to sequence complementarity and base pairing
12 Seq regions	Undefined sequences between the TRS regions, these often correspond closely to the ORF regions
3′UTR	5′ untranslated region

In this study, auditory streams were paired and played as stereo layers. Audio that plays consistently throughout the entire genome were played at low frequency and transient data was highlighted at a higher frequency register to make them more prominent. In addition to simply considering the basic construction of pitch and separation, the data was harmonised to make it more listenable. The root tone and third note of the scale were played across two octaves with the limited 4 note mono-nucleotide sonification to establish a strong harmonic landscape throughout the playback. The drone generated from the GC content (which is sometimes invariant for periods of time) was also used to reinforce the foundation of the basic scale harmony. The G or C bases, as nucleotide, di-nucleotide or trinucleotides were each matched to higher octaves and A and U were mapped to lower octaves. This was done consistently between these audio streams in an attempt to harmonise the otherwise random note selection based on sequence information. An exception to this principle was made for start and stop codons which were mapped to higher pitches than GC rich codons so that their occurrence was easily perceived in the auditory display (since higher pitched notes are perceived to be louder). Given that these codons are used to trigger and halt individual audio streams this approach further emphasises the occurrence of an open reading frame.

The wider note range of the codons (20 notes) were used to introduce leading tones that often sound more dissonant and add complexity to the harmonic spectrum. This allows them to be easily discerned above the landscape tones of the simpler motifs. Lastly, less frequent audio from dispersed regions of the genome e.g. TRS or stem-loop (SL) motifs were pitched at the highest octave ranges or more dissonant notes within the diatonic scale to highlight their occurrence. All of this was done within a mode of the diatonic major scale. Translation was played in Bb Aeolian (Bb, C, Db, Eb, F, Gb, Ab) whereas transcription was played in C Lydian (C, D, E, F#, G, A, B). The parameters for mapping of each RNA feature into an audio stream is summarised in Table 3. These choices are arbitrary and in later iterations of the tool it may be possible to choose the scale modes and key of choice. The Ionian mode mode of the major scale was avoided since this is generally considered to be happy sounding and inappropriate for the data.

**Table 3:** Scale degrees and instrumentation of the RNA features being sonified

Sonified motif	Instrument	Pan	Translation Scale Bb aeolian mode		Transcription Scale C Lydian mode	
			Scale degrees	Octave	Scale degrees	Octave
Nucleotide	Synth	L	1, 3	2, 3	1, 5	2, 3
Di-nucleotide	Synth	R	1, 4, 5, 6	1, 2, 3, 4	1, 3, 5	1
GC Content (10 bp)	AM synth + delay	L	1, 3, 6, 7	2,3	1, 3, 5, 7	4, 5
GC Content (100 bp)	AM synth + delay	R	1, 3, 6, 7	2, 3	1, 3, 5, 7	4, 5
3 bp repeat	Synth	L	1, 3	4	1, 4, 5	6
Codon Frame 1 (translation)	FM synth + distortion	L	1, 3, 4, 5, 7	2, 3, 4, 5	–	–
Codon Frame 2 (translation)	FM synth + distortion	C	1, 3, 4, 5, 7	2, 3, 4, 5	–	–
Codon Frame 3 (translation)	FM synth + distortion	R	1, 3, 4, 5, 7	2, 3, 4, 5	–	–
Tri-nucleotide (transcription)	FM synth + distortion	L	–	–	1, 3, 4, 5, 7	3, 4, 5
Untranslated regions	AM synth	R	1, 2, 3	5	1, 4, 6, 7	3
Transcription regulating sequences (TRS)	AM synth	L	1, 2, 4, 5, 6	5	1, 2, 3, 4, 5, 6, 7	6
Cleavage sites in the polyprotein	AM synth + distortion	L	1, 6, 7	4	1, 2, 3	6
Stem and loop regions (SL)	AM synth + delay + distortion	R	1, 2, 6, 7	5	1, 4, 5, 7	

Each nucleotide generates a note on every beat whereas each di-nucleotide generates a note every second beat. Each codon (in an ORF) generates a not every third beat. Together these notes are syncopated to create a characteristic sound during peptide translation that is distinct from the surrounding untranslated region. Audio from the GC tracks are only triggered when the GC ratio changes by an increment of 0.1. If a note sequence has identical adjacent notes then the length of the note is extended rather than being repeated. This creates space and clarity for other notes layered in the auditory display.

Translation of the genomic RNA leads to expression of a large polyprotein following ribosome binding to the 5′ prime untranslated region. However, from this genomic template the subsequent genes downstream from the polyprotein cannot be directly expressed presumably due to the stop codon at the end of the gene. In the display the sonification also stops at this point, however, play can be resumed to inspect the downstream sequence. Additionally, the tempo of the untranslated regions are slower than that of the coding regions so that the tempo increases as the play-head (in place of the ribosome) reads into a gene sequence. This was implemented to help the user distinguish between different sequence types during the display of translation.

One of the more interesting characteristics of the viral genome is the phenomena of discontinuous transcription whereby a template switch occurs during the synthesis of sub-genomic negative-strand RNA’s [5]. Various mechanisms have been proposed to explain how the transcription regulatory sequences (TRS) are involved in the synthesis of positive strand sub-genomic RNA from various negative strand intermediates [34]. TRS sequences are located in the untranslated regions between the genes and one model suggests that these facilitate transcription skipping to the TRS sequence located in the 5′ untranslated region. This process is driven by complementary interactions between TRS regions to add a copy of the leader sequence to form sub-genomic RNA species. In these sub-genomic RNA’s the polyprotein sequence has been omitted and ribosome binding at 5′ end can read through and express the contiguous downstream gene sequence [35]. This functional behaviour of the RNA has been built into the auditory and visual display. By default, the process of auditory translation runs from the 5′ end through to the stop codon at the end of the polyprotein, whereas transcription runs the full length of the RNA beginning at the 3′ end. A toggle switch, labelled ‘Translate as sub-genomic RNA’ has been implemented to change these behaviours. When the toggle switch is selected during the transcription mode, the play-head will skip from any upcoming TRS region to the leader TRS1 located in the 5′ region (mimicking the behaviour of the RNA replicase). Subsequently in translation mode with the toggle activated the play-head will, by way of example, skip from the leader TRS1 (omitting the polyprotein) through to the TRS2 region adjacent to the start of the S protein. Whilst the metadata use to drive this behaviour does not change the characteristics of the sound, it does change the selection which regions are sonified.

The website does not rely on a server and instead the entire RNA sequence is downloaded into the client browser when the page is loaded. All code is written in JavaScript and runs within the client browser. The React framework was used to create the environment state whereby each iteration of state represents a sliding window to the next base. Redux was also used to help manage state. Audio is generated in real time within the client browser using Tone.js. The Reactronica framework [36] was used to further manage audio within the environment state.

Translation of the viral polyprotein is known to be subject to a frameshift mutation and since this does not follow the normal rules of gene expression a conditional expression was used to change the display for that instance so that the translated protein in frame 2 shifts to frame 1 in both the visual and auditory display.

## Results and discussion

To understand the function of the viral plus RNA strand the information needs to be processed in the 5′ to 3′ direction during translation and in the reverse 3′ to 5′ direction for transcription (whereby nucleotides are extended to the newly synthesised minus strand at the 3′ end). In this study an auditory display of the sequence was generated with a sliding window moving in either direction. Processing of information within the sliding window was used to generate a synchronised auditory and visual display. This is advantageous since it mimics the behaviour of biological processes within the cell. To further emulate translation the generation of audio was triggered by start codons and silenced by stop codons. Furthermore, the visual display shows all possible peptide sequences and these are aligned with the RNA sequence being processed. From the sequence data alone the tool was able to detect and display all known open reading frames and metadata was used solely to label these in the display. Other open reading frames were detected throughout the genome in the displays, however, since these are not downstream of an in-frame ribosome binding site no claim is made that these are actually translated.

**Fig. 2:**
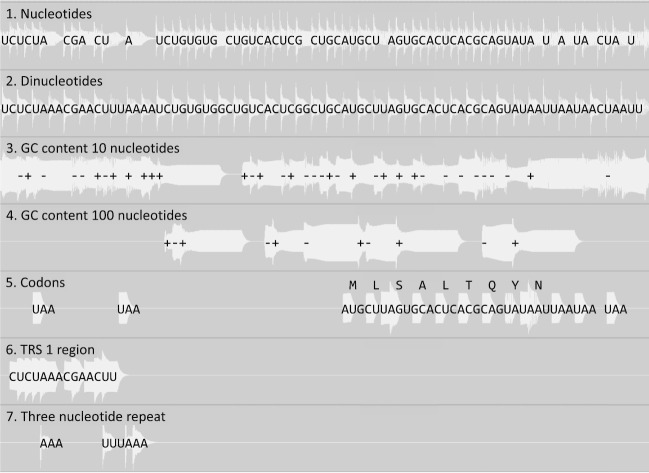
Multitrack wave files representing a portion of an auditory display. These tracks play in unison to generate the auditory display and each represent approximately 80 nucleotides beginning at nucleotide position 65. This sequence is located in the 5′ untranslated region and includes a TRS region and a uORF. Each audio stream was generated from a different algorithm, only nucleotides that gave rise to audio are shown (the entire nucleotide sequence is shown in track 2). In track 1, each nucleotide generates a note for every beat unless it is a repeat of the previous in which case the length of the note is extended. In track 2, each di-nucleotide generates a note every second beat. In tracks 3 and 4, audio from the GC track is only triggered when the GC ratio changes by an increment of 0.1. Each change in the GC ratio is indicated by a plus (+) or minus (−) symbol on the wave files. In track 5, only codon sequences beginning with a start codon (AUG) are shown through to the next stop codon (e.g. UAA). Isolated stop codons also give rise to a note. This track is a compilation of audio form three sub-tracks each representing a different reading frame and notes in this track are panned left, centre or right, respectively. Track 6 represents the audio generated from metadata that indicates the location of a TRS region. Additionally, the consensus sequence within this region is coloured purple in the visual display. Track 7 represents audio generated by the occurrence of three nucleotides of the same type. Other data tracks are not represented since no audio was generated in these during processing of this sequence of the genome. Additionally, the amino acid sequence of the ORF is shown in the codon track 5

High resolution analysis of gene expression in Coronavirus genomes has detected ribosome protected fragments which map to non-canonical ORF’s, these may be novel protein-coding ORFs and short regulatory uORFs. The tool highlights the occurrence of one such uORF of 30 nucleotides (including the stop codon) in the 5′ untranslated region downstream of TRS1 [35] that is not documented in the GenBank metadata. An image of the raw wave files and their relationship to the sequence information for this region are shown in Fig. 2. Non-standard uORF’s such as this have been detected as translation products in RNA sequencing and ribosome profiling experiments which allude to the complexity of gene regulation [37]. For this reason, all open reading frames are included in the display.

This uORF is clearly represented in Additiaonal file 1: Example 1, supplementary file ‘Sonification Untranslated ends’ (MP3 file) whereby at 26 s into the auditory display of the 5′ untranslated region a high-pitched start codon introduces a short sequence of layered audio that is punctuated a few seconds later by another high-pitched note as the layered audio ends. This can also be observed in Example 1 (MP4 file) as a nine amino acid residue sequence in reading frame 2. The 5′ untranslated region is also characterised by the distinctive sound of the TRS1 sequence at 16 to 19 s into the audio display. Similarly, three short ORF’s are apparent in the 3′ untranslated region of Example 1 beginning at 1 min 31 s following the high-pitched repetitive pattern of the SL region. These two untranslated regions were manually played one after the other during the same auditory display using the navigation buttons. Since they are both characterised by the absence of long open reading frames they provide a good introduction to the basic sound of the auditory display over which the highlighted notes from other RNA features will be layered.

**Fig. 3:**
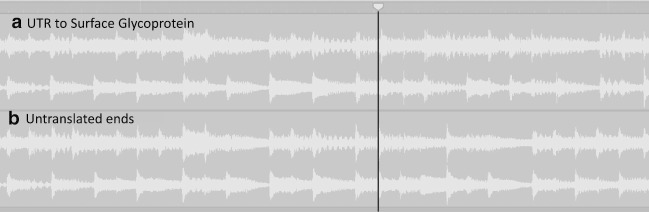
Alignment of the raw stereo waveforms. Two stereo waveforms are shown that depict the audio from examples 1 and 2. The vertical cursor indicates the transition across the TRS1 consensus sequence. Panel A depict the audio from the ‘UTR to Surface Glycoprotein’ example and panel B depicts that from the ‘Untranslated ends’ example. To the left of the cursor the stereo waveforms are identical leading up to the TRS1 region. To the right of the cursor the waveforms diverge. Panel A represents translation of a template produced through discontinuous transcription whereas panel B represents translation of contiguous genome sequence

The Additional file 2: Example 2 ‘Sonification UTR to Surface Glycoprotein’ (supplementary file) represents the sonification of a sub-genomic RNA. For this run the ‘Translate as sub-genomic RNA’ checkbox was selected to mimic translation from one of the products of discontinuous transcription, a process upon which viral gene expression is reliant. Sonification of the entire genome in either direction results in an auditory display lasting up to 2 h in duration. Selecting the ‘Translate as sub-genomic RNA’ checkbox results in a shorter auditory display since shorter regions of RNA are processed. Example 2 again plays from the beginning of the plus strand sequence (as does Example 1), however in this display the play-head skips from TRS1 to TRS2 and immediately into the ORF of the Surface Glycoprotein (skipping a portion of the untranslated region and skipping all of the ~21,000 bp of the polyprotein sequence). The display highlights that the prior discussed uORF is skipped in the 5′ leader of the sub-genomic RNA from which the genes downstream of the polyprotein are translated. The audio diverges from Example 1 after 23 s or so since the layered sound of the Surface Glycoprotein (an open reading frame) begins and continues to play for the rest of the display. Portions of the two stereo waveforms of the display from Example 1 and 2 are shown in Fig. 3. To the left of the cursor both stereo waveforms are essentially the same whereas to the right of the cursor the audio displays have clearly diverged as different sequences were processed beyond TRS1.

The Additional file 3: Example 3 ‘Sonification of the Nucleocapsid Phosphoprotein’ further builds upon the two prior examples. This supplementary file example begins with the Nucleocapsid Phosphoprotein gene and a clear duplet note pattern can be heard that is characteristic of two open reading frames playing simultaneously. The associated visual display shows that this pattern continues for approximately 100 amino acid residues. Whilst this may only be an artefact of the analyses rather than an undocumented protein sequence it does demonstrate the auditory display is capable of detecting unusual features in the genome. It is also worth noting that frame shift mutations do occur in the polyprotein sequence through a process that is not fully understood giving rise to a protein sequence that does not follow canonical gene expression patterns. The tool highlights the position of other relatively long open reading frames within the display so that they can be considered in the analysis of genome function. The Nucleocapsid Phosphoprotein sequence is followed by the ORF 10 sequence which is about one third the length of this parallel ORF. This analysis also highlights one of the properties of the auditory display which is that data in the three possible reading frames give rise to a triplet note pattern whereas data in two reading frames gives rise to a duplet note pattern (e.g. from 1 min 9 s through to 1 min 17 s). These note patterns make it easier to distinguish the features in the auditory display. In the last 1 min and 30 s of the display the genome alternates between gene sequences, transcription regulatory sequences, ORF10, stem loop structures and untranslated regions. These features have been further annotated in the video file with circles and arrows to emphasise their occurrence in the combined visual and auditory display.

In the Additional file 4: supplementary example ‘Sonification Sub-genomic RNA’ the auditory display represents the process of transcription. The tempo and scale patterns used for these displays are distinct from those used to represent translation. Additionally, no attention was paid to the occurrence of open reading frames or codon usage patterns since these pay no role in genome replication or transcription. Metadata relating to SL and TRS elements were sonified, however, cleavage information relating to the poly-protein modification did not seem relevant. The resulting auditory display is therefore simpler than that arising from translation. This can be heard in Example 4 which begins with sonification of the poly-A tail. In this example the play-head skips from TRS10 through to TRS1 which models the behaviour of discontinuous transcription. There is a check box on the page to switch between normal genome replication (whereby the entire genome would be sonified) and discontinuous transcription.

The Additional files 5, 6 and 7: Examples 5 to 7 in the supplementary files include regions already describe in the previous auditory displays. However, in these examples various streams of audio that contribute to the auditory display have been toggled on and off. Checkboxes are provided on the web page to facilitate this on the left-hand side of the note display table. The reason for this is two-fold. It provides a method to delineate how each feature of the RNA genome contributes to the auditory display. For instance, the sound of a TRS element or open reading frames could be highlighted (soled) or excluded (muted) from the overall sound of the translation display. This provides a better understanding of how the auditory displays are constructed. Secondly for those who are less interested in the science of Coronavirus and who are more interested in algorithmic music generation these tools can be used to compose and modify the inherent audio stream. The first of these, example 5 ‘Remix UTR through to Polyprotein’, highlights the contribution that GC content makes to the audio stream since these features are soloed at the beginning of the display. At one minute into the display audio from the translated amino acids are also toggled on or off to highlight their contribution. Example 6 ‘Remix ORF10 to the poly-A tail’ highlights the off-beat syncopation between the dinucleotides playing every second beat against codons playing every third beat. Lastly Example 7 highlights how important it is to continually sonify individual nucleotides across the sequence, since this provide a sonic landscape to overlay the other features. To emphasise this the individual bases were soloed at the beginning and excluded at the end of the display. All previously mentioned example files have been uploaded as supplementary files.

In addition to using the tool to navigate and inspect the function of the genome, the tool has been used to generate isolated audio in both translation and transcription modes. A playlist of four tracks has been uploaded to SoundCloud. These audio tracks are to be listened to without reference to the visual display. The intention of this is to engage the non-specialised community with the concept of ‘the sound of the Coronavirus genome’ and hopefully encourage people to delve a little deeper into the ideas behind the concept. Without the context of the display and without a clear understanding of the molecular biology of RNA virus the audio has to engage purely on its own sonic qualities—as an example of algorithmic music. In translation mode two auditory displays were prepared, the first (Covid-19 Translation polyprotein) plays through to the end of the polyprotein lasting 1 h and 8 min, covering approximately 21,500 nucleotides. The second audio track from a sub-genomic RNA (Covid-19 Translation discontinuous) skips the polyprotein entirely to the untranslated region prior to the Surface glycoprotein and then plays through to the 3′ poly-A tail. This piece covers approximately 8500 nucleotides and lasts 27 min. In addition, two audio tracks were generated representing transcription/ RNA synthesis. The track representing genome replication (Covid-19 Transcription) last 2 h. The track representing discontinuous transcription (Covid-19 Transcription discontinuous) skips between TRS10 and TRS1 lasts only 1 min and 47 s.

## Conclusion

This paper extends prior work whereby DNA was sonified using the rules of gene expression to generate an auditory display. Previously an individual algorithm was used to produce an individual stream of audio from either a nucleotide, a di-nucleotide or codons and it was concluded that the sonification of codons was the most useful to identify mutations in repetitive DNA or to distinguish coding regions from non-coding regions [6]. Here we layer up to 12 layers of audio, each relating to an RNA feature of interest. These include metadata to layer RNA features such as consensus sequences in TRS regions, SL regions, cleavage sites in the polyprotein and interspersed untranslated regions between characterised ORF’s. This approach produces a more detailed and rich auditory display which acts as a viable complement to an animated visual display.

Metadata was also used to affect the behaviour of the display to mimic what is known to occur during the Coronavirus life cycle. The polyprotein is the only product to be translated from the genomic RNA since this is thought to be the only ORF that has access to the ribosomal binding site in the 5′ untranslated region. To mimic this the default behaviour of the tool is to stop at the in-frame stop codon at the end of the polyprotein. The tool can be restarted at the adjacent untranslated region or elsewhere using the navigation buttons. The default behaviour in transcription mode is to read through the entire genome sequence from end to end to mimic genome replication to produce the complementary minus strand. A toggle switch has been implemented to mimic discontinuous transcription and in the first instance the polymerase will jump from TRS10 to TRS1 in the 5′ leader region. This can be overridden using the navigation buttons but if another TRS region is encountered by the play-head it will also jump to the TRS1 in the leader region. In translation mode the same toggle causes the ribosome play-head to skip the polyprotein and skip from TRS1 to TRS2 and into the Surface Glycoprotein sequence. From this point the play-head will continue to the 3′ end reading through the remainder of the genome. All other stop codons will be sonified but they will not influence the progression of the play-head. The auditory display in combination with real-time animation provide a unique insight into the large body of evidence describing the metabolism of the RNA genome. This provides another useful tool in the domain of genome browsers to further understand the complex function of the viral genome.

## Availability and requirements

*Project name* Real-time audio and visual display of the COVID-19 genome.

*Project home page* https://coronavirus.dnasonification.org/.

*Operating system*: Platform independent.

*Programming language* JavaScript (ECMAScript 6).

*Other requirements* None.

*License* GNU GPLv3.

Any restrictions to use by non-academics: No restrictions.

## Supplementary information

Additional file 1. Example 1 Sonification Untranslated ends.

Additional file 2. Example 2 Sonification UTR to Surface Glycoprotein.

Additional file 3. Example 3 Sonification of the Nucleocapsid Phosphoprotein.

Additional file 4. Example 4 Sonification Sub-genomic RNA.

Additional file 5. Example 5 Remix UTR through to Polyprotein.

Additional file 6. Example 6 Remix ORF10 to the poly-A tail.

Additional file 7. Example 7 Remix Membrane Glycoprotein.

## Data Availability

Source code is available on https://github.com/markTemple/coronavirus-sonification. Audio tracks generated by the tool for a non-specialised audience are available on a SoundCloud playlist. This playlist includes four tracks. These are, Covid-19 Translation polyprotein, Covid-19 Translation discontinuous, Covid-19 Transcription, and Covid-19 Transcription discontinuous. This playlist is available on https://soundcloud.com/templemark/sets/the-sound-of-the-coronavirus.

## Abbreviations

SL: Stem-loop
TRS: Transcription regulatory sequences
ORF: Open reading frame
